# Examination of the leaf proteome during flooding stress and the induction of programmed cell death in maize

**DOI:** 10.1186/1477-5956-12-33

**Published:** 2014-06-11

**Authors:** Yu Chen, Xi Chen, Hongjuan Wang, Yiqun Bao, Wei Zhang

**Affiliations:** 1 Department of Biochemistry and Molecular Biology, College of Life Science, Nanjing Agricultural University, Nanjing, Jiangsu, PR, China

**Keywords:** Maize, Programmed cell death, Flooding, Two-dimensional fluorescence difference gel electrophoresis, Translationally controlled tumor protein, S-adenosylmethionine synthase 2, Polyamine

## Abstract

**Background:**

Maize is a major economic crop worldwide, with substantial crop loss attributed to flooding. During a stress response, programmed cell death (PCD) can be an effective way for plants better adapt. To identify flooding stress related PCD proteins in maize leaves, proteomic analysis was performed using two-dimensional fluorescence difference gel electrophoresis (2D-DIGE) and mass spectrometry.

**Results:**

Comparative proteomics was combined with physiological and biochemical analysis of maize leaves under flooding stress. Fv/Fm, qP, qN and relative water content (RWC) were found to be altered in response to flooding stress, with an increase in H_2_O_2_ content noted *in vivo*. Furthermore, DNA ladder detection indicated that PCD had occurred under flooding treatment. The maize leaf proteome was analyzed via 2D-DIGE gel, with a total of 32 differentially expressed spots isolated, 31 spots were successfully identified via MALDI-TOF/TOF MS which represent 28 proteins. The identified proteins were related to energy metabolism and photosynthesis, PCD, phytohormones and polyamines. To better characterize the role of translationally controlled tumor protein (TCTP) in PCD during a stress response, mRNA expression was examined in different plants by stress-induced PCD. These included heat stress induced rice protoplasts, Tobacco Mosaic Virus infected tobacco leaves and dark induced rice and *Arabidopsis thaliana* leaves, all of which showed active PCD, and TCTP expression was increased in different degrees. Moreover, S-adenosylmethionine synthase 2 (SAMS2) and S-adenosylmethionine decarboxylase (SAMDC) mRNA expression were also increased, but ACC synthase (ACS) and ACC oxidase (ACO) mRNA expression were not found in maize leaves following flooding. Lastly, ethylene and polyamine concentrations were increased in response to flooding treatment in maize leaves.

**Conclusions:**

Following flooding stress, the photosynthetic systems were damaged, resulting in a disruption in energy metabolism, with the noted photosynthetic decline also possibly attributed to ROS production. The observed PCD could be regulated by TCTP with a possible role for H_2_O_2_ in TCTP induction under flooding stress. Additionally, increased SAMS2 expression was closely associated with an increased polyamine synthesis during flooding treatment.

## Background

Programmed cell death genetically controls the rate of cell division and death to strictly regulate cell numbers in both animals and plants, thus ensuring that cells that are no longer needed can activate their own demise [1]. Studies have indicated that PCD cannot be limited to the regulation of development or reproduction, but is also implicated in plant senescence [2,3] and other process such as defense against biotic [4] and abiotic [5,6] stresses.

Flooding, a major abiotic stress, poses as a major constraint affecting crop growth, production and productivity in many agricultural regions worldwide [7]. The soil is considered to be flooded if there is freestanding water on the soil surface or if the available water fraction of the surface layer is at least 20% higher than the field capacity [8]. Maize is an important economic crop, with an estimated worldwide production of 839 million tons according to World Agricultural Supply and Demand Estimates report from October 11, 2012. However, in Southeast Asia, approximately 15% of the maize growing areas are affected by flooding, resulting in yearly production losses ranging from 25% - 30%. In southeastern China, heavy rainfall leads to flooding that generally occurs during the maize seedling stage, resulting in severe seedling damage reducing maize production. While flooding is becoming a growing concern worldwide in numerous agriculture areas [9], recent evidence indicates that flood-induced PCD is related to aerenchyma formation and endogenous ethylene synthesis in maize [10].

To further characterize the molecular mechanisms regulating PCD in maize, proteomic analysis was employed. While proteomic approaches have been effective in characterizing protein expression patterns during stress responses [11], only a few proteomic studies have examined flooding or anoxia stresses in plants, which include tomato [11], rice [12], soybean [13], wheat [14] and maize [15]. Previous studies have indicated that an early rise in cytosolic Ca^2+^, an establishment of ionic homeostasis and root tip death may be essential adaptive changes enabling flood tolerance in maize [16]. To our knowledge, no proteomic study of maize leaf PCD during flooding has been examined. Our results indicated that following 4-days (4d) of flooding treatment, a conspicuous DNA ladder was noted in the third leaf, indicating the occurrence of PCD. Subsequently, third leaf total protein extracts were analyzed via two-dimensional fluorescence difference gel electrophoresis (2D-DIGE), and 28 proteins relating to energy metabolism/photosynthesis, PCD, phytohormones and polyamine-responsive proteins were identified. Furthermore, amongst various stresses including heat induced stress in rice protoplasts, Tobacco Mosaic Virus (TMV)-infected tobacco leaves and dark induced stress in rice and *Arabidopsis thaliana* leaves, all led to a relative increase in translationally controlled tumor protein (TCTP) mRNA expression and showed active PCD.

## Results and discussion

### Physiological measurements, *in vivo* H_2_O_2_ accumulation and induction of PCD in maize leaves during flooding

**Figure 1:**
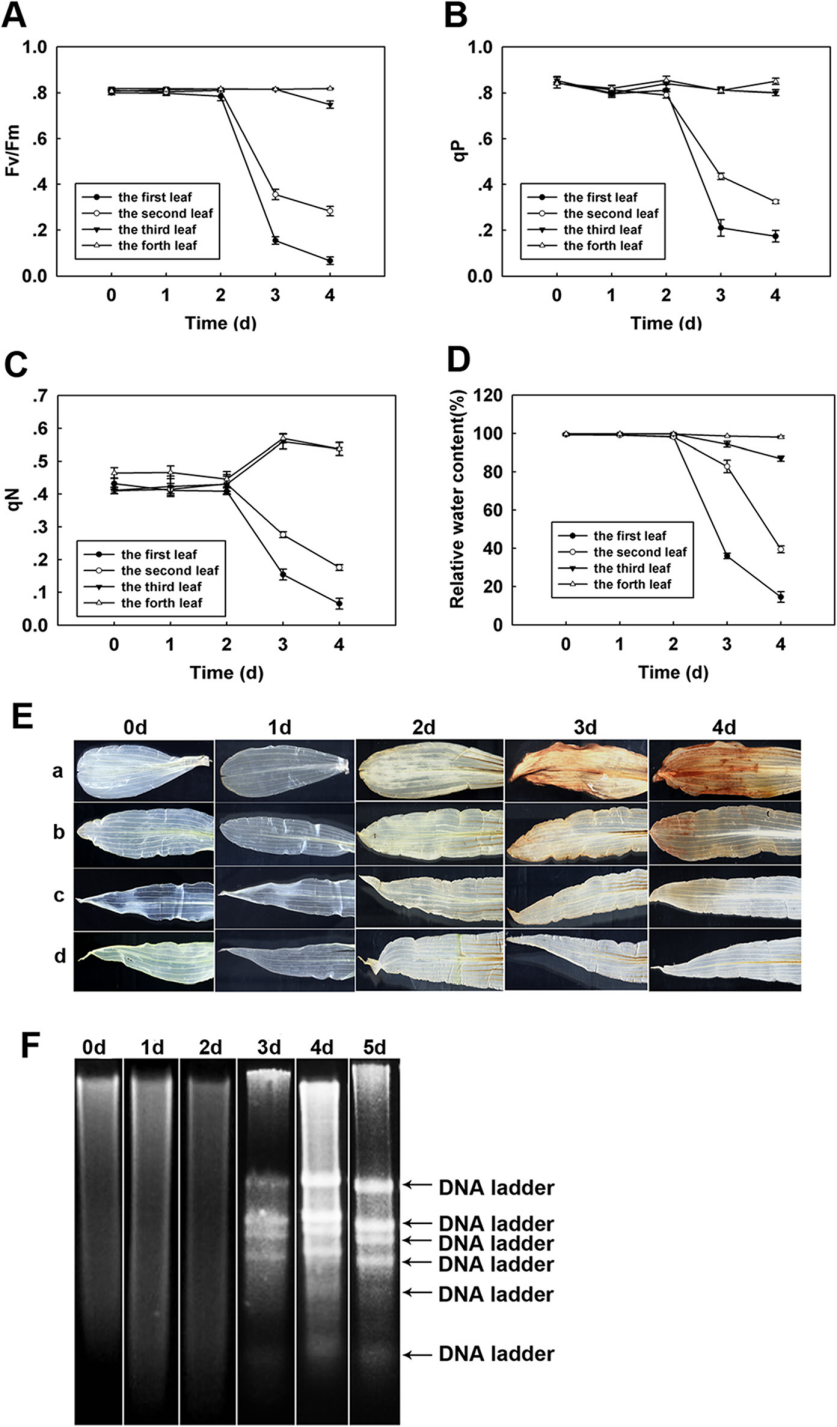
**Physiological and biochemical analyzed maize leaves under flooding treatment.** Comparisons of means of total Fv/Fm ratio **(A)**, qP **(B)**, qN **(C)** and leaf relative water content (RWC) **(D)** under flooding treatment with all four leaves for 0, 1, 2, 3, 4 d. Vertical bars represent means ± SD (n = 4) where these exceed the size of the symbol, as determined by Fisher’s protected LSD test (p < 0.05). Time-course analysis of H_2_O_2_ production in maize leaves exposed to flooding treatment for 0, 1, 2, 3, 4 d, to include the first leaf **(a)**, second leaf **(b)**, third leaf **(c)** and fourth leaf **(d) (E)**. DNA laddering after different treatment times with flooding for 0, 1, 2, 3, 4, 5 d **(F)**.

To study the role of maize physiology during flooding treatment, the maximum quantum yield of PSII (Fv/Fm), the photochemical quenching coefficient (qP), the non-photochemical quenching coefficient (qN) and leaf relative water content (RWC) were analyzed in different leaves during various durations of treatment. After 2 days of flooding exposure, all of these factors showed little affected. However, after 3 days of treatment, Fv/Fm, qP and RWC were decreased in the first, second and third leaves, with a lesser change noted in the fourth leaves (Figure 1A,B,D). Moreover, the qN value was decreased in the first and second leaves after 3 days of flooding treatment and yet was increased in the third and fourth leaves, with no decrease noted in these leaves until reaching 4 days of treatment (Figure 1C). In Sorghum exposed to flooding stress, Fv/Fm and qP significantly decreased, but qN increased substantially under saline conditions [17]. The noted Fv/Fm ratio decrease indicates a down regulation of photosynthesis, or photoinhibition [18], and the relatively low leaf water content clearly predisposes the leaves to photoinhibitory damage [19]. The noted decreased in qP is considered indicative of a down-regulation of electron transport [20] and the increase in qN reflects a reduced demand for electron transport products and an increased heat dissipation [21]. Furthermore, a maintenance or increase in qN values in stress situations has been associated with a protective response in order to avoid photoinhibitory damage to the reaction centres [22]. These findings suggested that after 4 days of flooding treatment, the photosynthetic systems of the first and second leaves were damaged and affected the third and fourth leaves. To investigate flood-induced H_2_O_2_ production, histochemistry utilizing 3, 3-diaminobenzidine (DAB) staining was employed. During DAB staining, the DAB reacts with H_2_O_2_ in a POX-dependent *in situ* histochemical reaction producing a red-brown polymerization product. This showed H_2_O_2_ accumulation following 3 days of flooding treatment, with more conspicuous accumulations noted after 4 days of treatment, with DAB coloration mainly observed on the surface of the first and second leaves and in the tips of the third leaves (Figure 1E). Previous studies have noted that when plants are subject to stress, such as plum pox viral (PPV) treatment, the redox balance can be easily disturbed and ROS accumulation in chloroplasts, probably by a disturbance of the electron transport chain [23]. In the present study, flooding induced changes in the Fv/Fm, qP, qN and H_2_O_2_ levels, suggesting a decline in photosynthetic processes, possibly attributed to ROS production. As shown in Figure 1F, DNA laddering occurred following 3 days of flooding treatment, becoming more pronounced after 4 days of treatment and remained relatively the same after 5 days of treatment. The observed DNA laddering indicated the occurrence of PCD, with the 3 day samples exemplifying the early stage of PCD, with the DNA laddering further elevated by day 4. Since this research was focused on the execution of PCD, the third leaf following 4 days of treatment was used for PCD characterization. Thus, flooding stress can contribute to a change in RWC, Fv/Fm, qP, and qN. Moreover, an accumulation of ROS can occur, to include H_2_O_2_ accumulation, leading to leaf senescence [24]. Additionally, the DNA ladder was assayed in the third leaves, suggesting that under flooding stress PCD may occur at the early stages [25].

### Proteomics: identification of differentially expressed proteins

**Figure 2:**
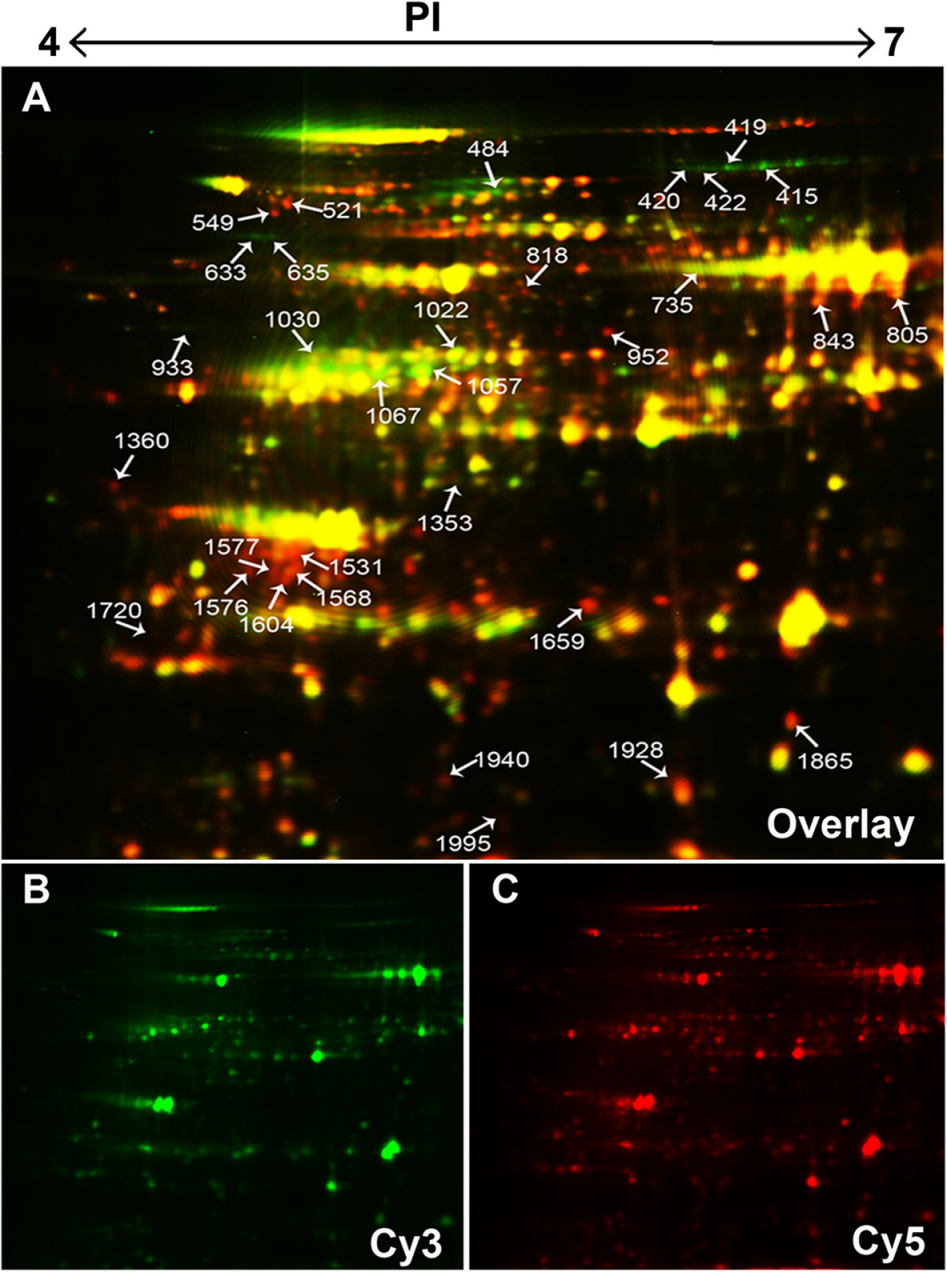
**2D-DIGE images of total leaf proteins from the control and flooding treatments in maize.** Extracts from control and treatment samples for four independent biological repeat experiments were differentially labeled with the Cy3 and Cy5 and separated by two-dimensional electrophoresis on 13-cm (pH 4–7) IPG strips and 12.5% polyacrylamide gels. Arrowed and numbered spots are differentially expressed protein spots in the image **(A)**. Cy3-labeled control **(B)** and Cy5-labeled treatment **(C)** is shown.

**Figure 3:**
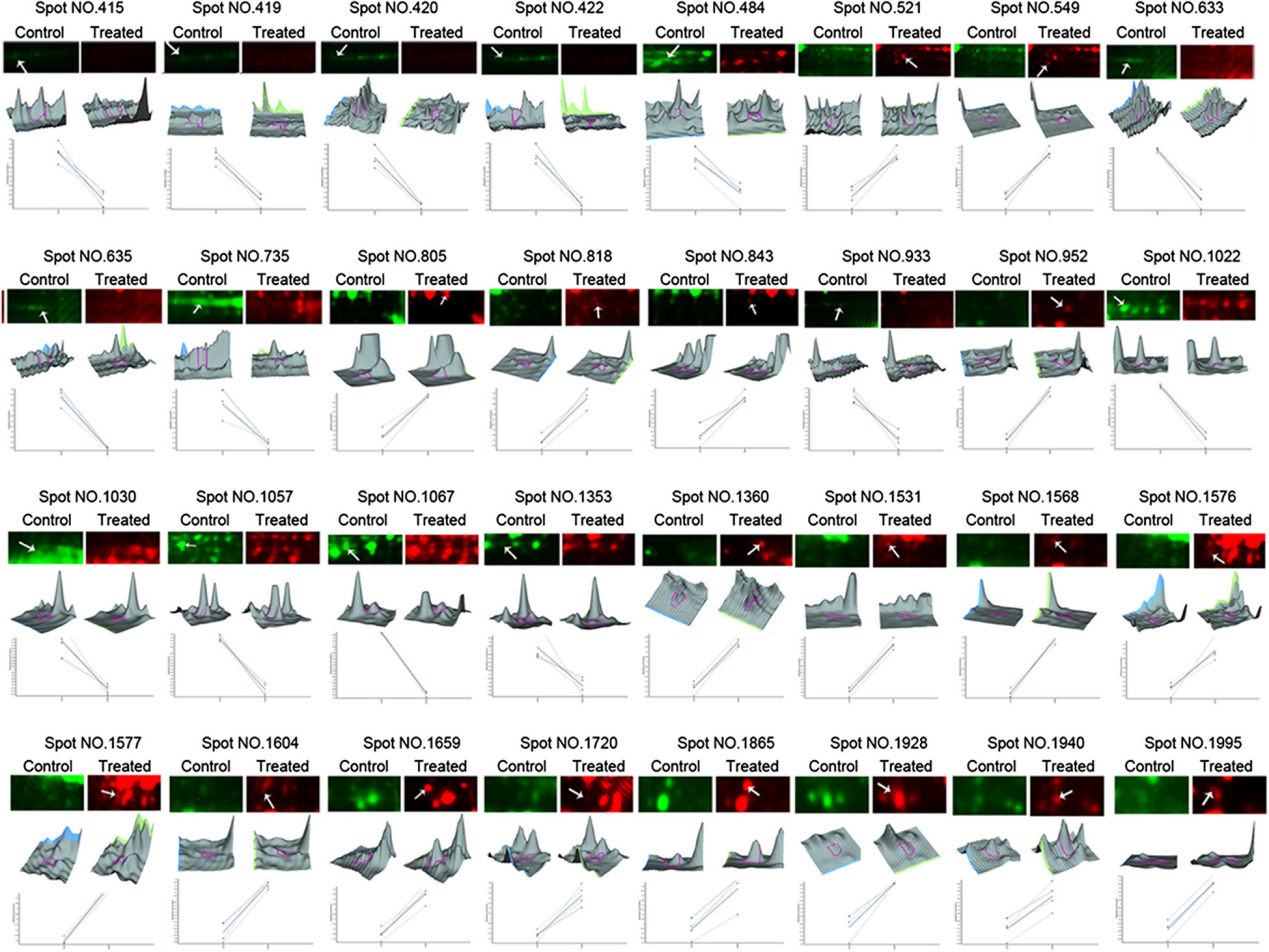
**Analysis of all identified proteins.** The readout of the DeCyder Biological Variation Analysis (BVA) module is shown for all identified spots. The 2D-DIGE gels for Cy3-labeled control (green) and Cy5-labeled treated (red) and the corresponding 3D views are shown. The graphic at the bottom panel shows the differences in abundance of these proteins across four independent experiments.

Equal amounts of protein from the control and treated leaved were labeled with Cy2 (internal standard), Cy3 or Cy5 dyes, with an overlay of the Cy3 and Cy5 images from the 2D-DIGE gels shown in Figure 2. In general, the protein expression patterns of treated samples were similar to those of the control leaves, with more than 2000 spots observed by the DIGE methodology. Following 2-DE image analysis, we found a number of spots with lower or higher protein abundances relative to the control leaves. A 2.5-fold threshold limit was set in this study, with four replicates performed to reduce the number of potential false positives. Figure 2 shows a representative DIGE image of control and treated leaf protein extracts labeled with Cy3 and Cy5 and separated with IPG 4–7 strips and the spots used for mass spectrometry analysis numbered, with some of these differential spots shown in the extended portion of the gels in Figure 3. Thirty-two spots showed at least a 2.5-fold change in protein abundance (p < 0.05) with 18 polypeptides exhibiting an increased expression and 14 polypeptides showing a decreased expression in the treated leaves relative to the control.

### Protein identification

The 32 differentially expressed spots were further analyzed using MALDI-TOF/TOF MS analysis, with 31 spots were successfully identified by searching against the NCBInr and Viridiplantae EST databases (Table 1) which represent 28 proteins. According to NCBI gene annotations and the literatures, these proteins could be functionally classified into various groups, including energy metabolism and photosynthesis (41.9%), PCD (32.3%), phytohormones and polyamines (16.1%) and others (9.7%). Of the proteins relating to energy metabolism/photosynthesis, a protein related to IAA metabolism (spot 484) and a protein related to GA induced expression (spot 1995) from the α*-Amy1* and α*-Amy2* promoters [26] were identified. Moreover, proteins associated with ethylene metabolism (spots 1353 and 843) and the S-adenosylmethionine synthase 2 (spot 952) protein, which is involved in both ethylene and polyamine synthesis and relates to stress response, were also identified. Furthermore, 3-beta hydroxysteroid dehydrogenase/isomerase family protein (spot 1604), chitinase (spot 1360), harpin binding protein (spot 1568), pleckstrin homology domain containing, family A (spot 1928), developmentally regulated plasma membrane polypeptide 4 (DREPP4, spot 1576), fruit protein PKIWI502 (spot 1577), heat shock protein 70 (spot 521, 549) and putative heat shock protein translationally controlled tumor protein (TCTP, spot 1720) were all shown to be related to PCD. Therefore, these results indicate that flooding can affect energy metabolism/photosynthesis, phytohormones and polyamines, possibly leading to the induction of PCD.

**Table 1:** Flooding treated responding proteins from maize leaves were analyzed by 2D-DIGE and MALDI-TOF/TOF

**Spots no.**	**Category and name**	**Gi number**	**TheroPI/Mr**	**Peptides matched**	**Coverage (%)**	**Score**	**S.V.R. (Treated/Control)**	***p-Value***
**Photosynthesis and energy metabolism related proteins**								
**415**	Phosphoenolpyruvate carboxykinase [ATP] [Zea mays]	gi|162457930	6.57/73781	3	6	66	-3.29	0.00034
**419**	Phosphoenolpyruvate carboxykinase [ATP] [Zea mays]	gi|162457930	6.57/73781	3	6	92	-3.06	0.00012
**420**	Phosphoenolpyruvate carboxykinase, putative, expressed [Oryza sativa Japonica Group]	gi|108707241	7.14/74523	2	4	58	-3.17	0.0029
**422**	Phosphoenolpyruvate carboxykinase [Zoysia japonica]	gi|58036453	6.49/72407	2	1	56	-3.34	0.0021
**633**	β-amylase	gi|1703302	4.88/55487.3	8	8	155	- 4.58	2.30E-05
**635**	RuBisCO subunit binding-protein alpha subunit, chloroplast precursor (60 kDa chaperonin alpha subunit)	gi|115488160	5.12/61151	2	5	87	-3.09	0.00087
**735**	ATPase subunit 1 (mitochondrion) [Zea mays subsp. parviglumis]	gi|102567957	5.85/55430.9	18	13	249	-2.58	0.013
**933**	Fructose-1,6-bisphosphatase [Zea mays]	gi|226498474	5.07/44512.36	4	12	185	-3.2	0.00023
**1022**	Malate dehydrogenase 1 [Zea mays]	gi|195612678	6.49/47385	4	10	253	-3.69	1.10E-05
**1030**	Phosphoglycerate kinase [Zea mays]	gi|223975935	5.21/43227	9	18	344	-2.52	0.00065
**1057**	Ribulose bisphosphate carboxylase/oxygenase activase, chloroplastic precursor [Zea mays]	gi|162458161	6.29/48079	5	16	275	-6.11	7.20E-06
**1067**	Adenosine kinase [Zea mays]	gi|4582787	5.23/36465.5	11	4	179	-10.45	5.10E-06
**1531**	Oxygen-evolving enhancer protein 1 [Zea mays]	gi|195619530	5.59/34783	2	8	72	5.31	6.60E-06
**1659**	50S ribosomal protein L21 [Zea mays]	gi|195609236	4.66/13133.9	3	17	92	2.57	0.00037
**PCD related proteins**								
**521**	Heat shock protein 70 [Arabidopsis thaliana]	gi|6746592	5.13/71056.4	2	4	67	3.46	0.00056
**549**	Heat shock protein 70 [Arabidopsis thaliana]	gi|6746592	5.13/71056.4	2	3	68	4.09	4.70E-05
**1360**	Chitinase [Zea mays subsp. parviglumis]	gi|214015047	4.88/34474.7	6	8	171	2.89	3.00E-05
**1568**	Harpin binding protein 1 [Oryza sativa Indica Group]	gi|38679325	8.92/28360.16	2	5	73	3.18	0.00013
**1576**	DREPP4 protein [Zea mays]	gi|195638402	4.89/22595.9	9	31	203	2.6	0.00078
**1577**	Fruit protein PKIWI502 [Zea mays]	gi|195624268	6.62/31174.73	4	20	154	3.15	0.0001
**1604**	3-beta hydroxysteroid dehydrogenase/isomerase family protein [Zea mays]	gi|195642948	7.63/32772.2	4	20	150	5.17	0.00015
**1720**	Translationally-controlled tumor protein [Zea mays]	gi|195605582	4.52/18773.4	5	6	88	2.52	0.00053
**1928**	Pleckstrin homology domain containing, family A [Zea mays]	gi|195641188	6.52/22785.4	7	9	53	3.4	0.006
**Phytohormone and polyamine related proteins**								
**484**	Aux/IAA protein [Solanum tuberosum]	gi|25989504	7.82/37018.4	11	15	73	-2.58	0.0032
**843**	Controlling leaf angle [Zea mays]	gi|343781534	4.74/43900.4	10	10	55	2.71	0.0036
**952**	S-adenosylmethionine synthase 2	gi|127046	5.57/43618	1	3	87	3.39	0.0006
**1353**	PISTILLATA-like MADS box protein [Crocus sativus]	gi|78146198	9.26/24915.8	11	9	64	-3.2	0.0057
**1995**	HRT transcription factor [Zea mays]	gi|323388729	9.71/60370.4	17	24	83	3.81	0.0046
**Other proteins**								
**805**	Unknown [Zea mays]	gi|194695026	6.52/42290.4	20	14	434	2.66	0.00056
**818**	Hypothetical protein [Zea mays]	gi|195612760	5.61/41336.1	5	7	128	2.61	0.0057
**1940**	OSJNBb0003B01.14 [Oryza sativa Japonica Group]	gi|58531981	6.02/192426.8	25	12	79	2.79	0.0095

### mRNA expressional analysis of differentially expressed proteins via qRT-PCR

**Figure 4:**
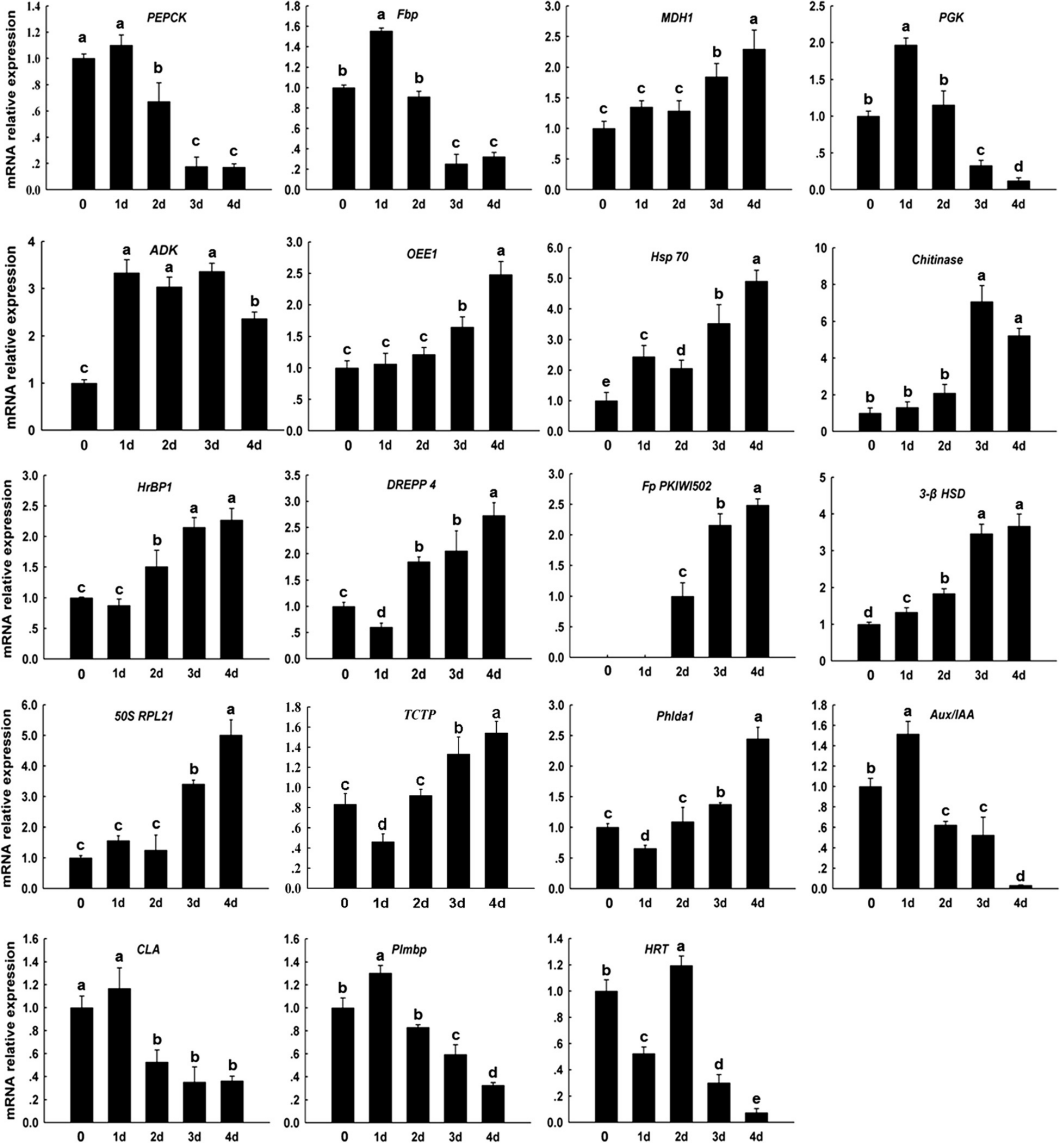
**Quantitative real-time PCR analysis of the mRNA expression levels of differentially expressed proteins under flooding treatment for 0, 1, 2, 3, 4 d.** PEPCK: phosphoenolpyruvate carboxykinase; Fbp: fructose-1,6-bisphosphatase; MDH1: malate dehydrogenase 1; PGK: phosphoglycerate kinase; ADK: adenosine kinase; OEE1: oxygen-evolving enhancer protein 1; Hsp 70: heat shock protein 70; Chitinase:Chitinase; HrBP1: Harpin binding protein 1; DREPP 4: DREPP 4 protein; Fp PKIWI 502: fruit protein PKIWI 502; 3-β HSD: 3-beta hydroxysteroid dehydrogenase/isomerase family protein; 50S RPL21:50S ribosomal protein L21; TCTP: translationlly-controlled tumor protein; Phlda: pleckstrin homology domain containing, family A; AUX/IAA: AUX/IAA protein; CLA: control leaf angle; Plmbp: PISTILLATA like MADS box protein; HRT: HRT transcription factor; Data are displayed as mean values ± SD from three independent experiments. The same letter indicated no significant difference, and different letters indicated significant differences, as determined by Fisher’s protected LSD test (p < 0.05).

Gene expression at the transcription level was examined in 19 identified proteins via qRT-PCR (Figure 4). When maize was subjected to moderate flooding stress, several genes such as *PEPCK, PGK, AUX/IAA*, *Plmbp, Hsp70, Chitinase* and *DREPP* were up-regulated [27]. Our results indicated that the mRNA expression levels of *PEPCK, Fbp, PGK, AUX/IAA, CLA, Plmbp* and *HRT* increased following 1 day of treatment, but showed a dramatic decrease in the subsequent treatments. Furthermore, twelve transcripts (*MDH1, ADK, OEE1, Hsp70, Chitinase, HrBP1, DREPP4, Fp PKIWI 502, 3-*β *HSD, 50S RPL21, TCTP* and *Phlda1*) showed increased expression levels following 4 days of flooding treatment. It has been suggested that low oxygen levels can lead to gene expression reprogramming to help the plant withstand stress, as well as to maintain photosynthesis, metabolism and complement auxin at optimum levels [27], whereas the severe stress may damage the photosynthetic system, disrupting energy and auxin metabolism. However, the transcriptional expression levels of four genes (*MDH1, ADK, CLA* and *HRT*) were different from the observed protein expression levels, making it insufficient to predict protein expression levels from quantitative mRNA data. This phenomenon has been observed in many other studies and is mainly due to the transcriptional, post-transcriptional, translational and post-translational events regulating expression [28-30].

### Photosynthesis and energy metabolism response of maize leaves during flooding stress

Rubisco is a major photosynthetic protein and is involved in the respiratory pathway in plants. In the present study, the RuBisCO subunit binding-protein alpha subunit (spot 635) and RuBisCO activase (spot 1057) which correspond to Rubisco were down-regulated. Analogous our results, down-regulation of the Rubisco-binding protein (chaperonin 60) and Rubisco activase have been previously reported under flooding conditions [31]. These findings may be key to the decreased chlorophyll content and photosynthetic rate, with some reports indicating that Rubisco is gradually degraded during leaf senescence [32-34], possibly indicating that flooding stress may accelerate leaf senescence. Phosphoglycerate kinase (spot 1030), participates in the Calvin cycle and catalyzes an ATP-dependent reaction to form 1,3-bisphosphoglycerate from phosphorylate 3-phosphoglycerate [35]. Down-regulation of this enzyme could indicate a decrease in photosynthetic carbon assimilation during flooding conditions. An up-regulation in oxygen-evolving enhancer protein 1 (OEE1, spot 1531), which is bound to photosystem II (PSII) on the luminal side of the thylakoid membrane and is the most important protein for oxygen evolution and PSII stability [36], was also noted. Some evidence suggested that OEE1 is involved in recovery/turnover, which maintains the capacity of PSII during salinity and drought stress [36-38], yet little evidence supports that OEE1 is associated with flooding stress. We postulate that the up-regulation of OEE1 might repair protein damage and keep oxygen evolving under flooding conditions. Additionally, the 50S ribosomal protein L21 protein (spot 1659) was also up-regulated and has been implicated in the transformation of proplastids to chloroplasts [39]. We postulate that as chloroplast damage occurs under flooding conditions and that the 50S ribosomal protein L21 protein could accelerate the transformation of proplastids to chloroplasts for survival. While ATP is an important energy source, ATP synthesis is low in the mitochondria during the oxygen deprivation that is experienced during flooding condition [40]. Adenosine kinase (ADK, spot 1067) is a housekeeping enzyme that catalyzes the phosphorylation of adenosine (Ado) into adenosine monophosphates (AMP) [41]. ADK and ATPase subunit 1 (spot 735) were found to be down-regulated, possibly due to an O_2_ and ATP reduced availability [42]. Other down-regulated proteins included β-amylase (spot 633), which is involved in the degradation of starch to sucrose [43], malate dehydrogenase 1 (spot 1022), phosphoenolpyruvate carboxykinase (spot 415, 419, 420, 422) and fructose-1, 6-bisphosphatase (spot 933) which all play a key role in gluconeogenesis. Collectively, these findings indicated that a lack of sugar production in hypoxic plants could restrict essential metabolites and in turn reduce energy consumption [42]. Additionally, sugar fermentation plays a key role in root tip acclimation during anoxia [15], with the current study noting an inhibition of enzymes pertaining to gluconeogenesis, thus enhancing fermentation.

### PCD response to flooding stress

**Figure 5:**
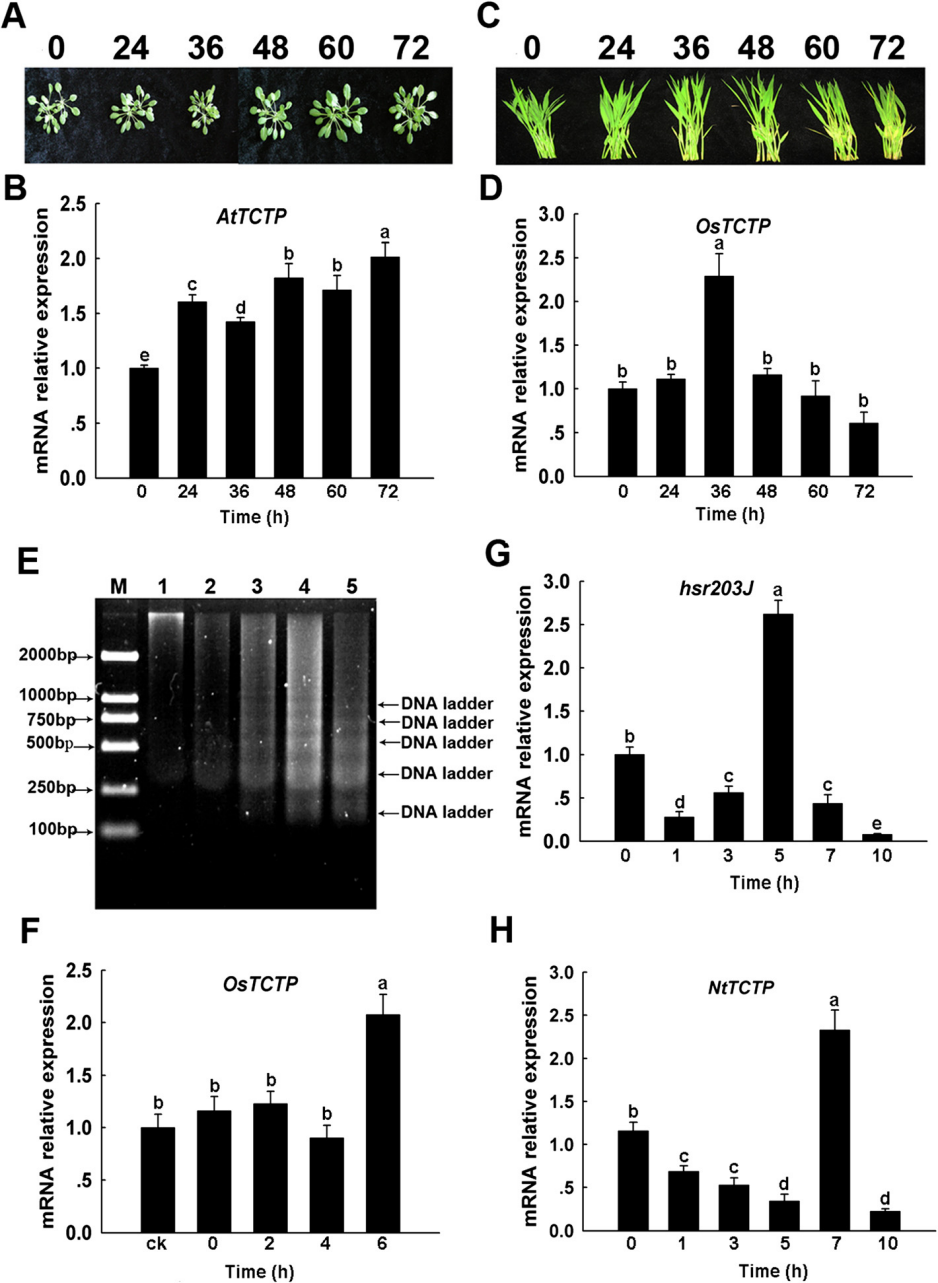
**Quantitative Real-time RT-PCR analysis of the mRNA expression levels of TCTP in various plants by PCD induction.** The morphology of Arabidopsis was induced by dark for 0, 24, 36, 48, 60, 72 h **(A)**. qRT-PCR analysis of *TCTP* mRNA expression levels in dark-induced Arabidopsis for 0, 24, 36, 48, 60, 72 h **(B)**. The morphology of rice was induced by dark for 0, 24, 36, 48, 60, 72 h **(C)**. qRT-PCR analysis of *TCTP* mRNA expression levels in dark-induced rice for 0, 24, 36, 48, 60, 72 h **(D)**. DNA laddering in rice protoplasts after heat treatment was detected by agarose electrophoresis. Protoplasts were treated at 48°C for 15 min and allowed to recover at 28°C for 0, 2, 4, 6 h. Lane M: Marker; Lane 1: untreated (ck); Lane 2: recovered for 0 h; Lane 3: recovered for 2 h; Lane 4: recovered for 4 h; Lane 5: recovered for 6 h **(E)**; qRT-PCR analysis of *TCTP* mRNA expression levels in heat shock rice protoplasts for ck, 0, 2, 4, 6 h **(F)**. qRT-PCR analysis for quantitative analysis of *hsr203J***(G)** and *TCTP***(H)** mRNA expression levels after TMV-infected tobacco for 0, 1, 3, 5, 7, 10 h. Data are displayed as mean values ± SD from three independent experiments. The same letter indicated no significant difference, and different letters indicated significant differences, as determined by Fisher’s protected LSD test (p < 0.05).

Plants subjected to biotic or abiotic stresses can experience PCD, while flooding conditions damaged crops due to anoxia, leading to an increased risk of plant disease and insect infestations [44]. Three proteins corresponding to diseases resistance, including 3-beta hydroxysteroid dehydrogenase/isomerase family protein (spot 1604), chitinase (spot 1360) and harpin binding protein 1 (HrBP1, spot 1568), were found to be up-regulated during flooding stress. A previous study found that following *R. solani* infection, the 3-beta hydroxysteroid dehydrogenase/isomerase family protein was induced in resistant rice, suggesting that this enzyme may play a role in the synthesis and regulation of steroids associated with disease resistance [45]. HrBP1 can induce plants to generate systemic acquired resistance and plays an important biological role in pest control [46], with its up-regulation able to induce resistance against viruses, fungi, bacteria and pests in plants [47]. Chitinase, a pathogenesis related (PR) protein, has been reported to strengthen the plant immune response against a variety of pathogens and has been noted to increase in response to numerous abiotic agents [48]. Additionally, previous studies have described the induction of PR proteins in response to both biotic and abiotic stresses, such as viral infection or salt stress treatment, and shown that PR induction can prevent opportunistic fungal or bacterial infections when the plant is in a weaken state [49-51]. Fruit protein PKIWI502 (spot 1577), who’s functions relate to FAD-dependent oxidoreductase, was also up-regulated. Oxidoreductase has been reported to show increased levels under flooding conditions [52], in addition to AIF, a FAD-dependent oxidoreductase, that regulates PCD [53]. Thus, we propose that fruit protein PKIWI502 could also regulate PCD. Pleckstrin homology domain containing, family A (Phlda1, spot 1928) was found to be up-regulated and has been associated with apoptosis in T cell hybridomas, neuronal and melanoma cells [54-56], but little has been reported in plants. DREPP4 (spot 1576) has been found to be a developmentally regulated plasma membrane polypeptide in tobacco [57]. DREPP-like protein, a calmodulin [58], was found to temporarily increase after cold acclimation [59], while DREPP 4 has been associated with defense response [60]. Additionally, an early rise in cytosolic Ca^2+^, as well as an establishment of ionic homeostasis was found to be essential for the induction of adaptive changes in response to flooding treatment in maize [16]. Moreover, changes in protein synthesis are required during hypoxia for improved cytoplasmic pH regulation and survival [15]. Together, these findings suggest that DREPP4 may play a key role in ionic homeostasis and be associated with Ca^2+^-mediated PCD. The up-regulation of heat shock protein 70 (hsp70, spot 521, 549) during flooding has been previously reported [15,61] and has been found to suppress PCD in rice protoplasts [62]. An increased expression of TCTP (spot 1720) has been reported under a variety of stress conditions in humans and *Mytilus galloprovincialis*, with TCTP functioning in the maintenance of heat stability and induction of cell death [63,64]. While little has been reported relating TCTP to PCD during maize stress response, our findings showed an up-regulation of TCTP during flooding in maize. Additionally, we analyzed TCTP transcriptional levels during stress induced PCD in various plants. Arabidopsis plants were incubated in the dark for 24, 36, 48, 60 and 72 h and showed an unaltered morphology (Figure 5A), while TCTP mRNA expression levels increased proportionately to the dark exposure duration (Figure 5B). Additionally, dark-induced rice experienced greater degrees of etiolation following exposure, with TCTP mRNA expression levels peaking at 36 h and then decreasing (Figures 5C and D). Rice exposed to heat stress showed DNA laddering, indicating the occurrence of PCD in rice protoplasts, with DNA laddering occurring at 2 h, becoming significant by 4 h and decreasing at 6 h (Figure 5E). TCTP mRNA expression levels were increased at 6 h relative to the control (Figure 5F). Hsr203j, the hypersensitivity (HR) molecular marker gene that accumulates specifically in tissues, was examined following TMV infection. This showed the *hsr203J* mRNA expression at 5 h to be approximately 2.6-fold higher than the control (Figure 5G), demonstrating that hypersensitivity (HR)-like cell death was induced by TMV. Furthermore, *TCTP* mRNA expression at 7 h was approximately 2.3-fold higher than the control. (Figure 5H). In Arabidopsis, PCD was shown to inhibit TCTP, with TCTP able to significantly diminish tunicamycin induced cell death and able to affect expression of the anti-apoptotic protein BAX [65]. These findings further suggest that TCTP plays a role in PCD during a stress response and TCTP may be a regulator of PCD in maize. In the present study, we observed an accumulation of H_2_O_2_ in leaves as a result of flooding stress (Figure 1E). In a previous study, H_2_O_2_ inhibition was found to induce TCTP protein expression in pea seeding [66], thus collectively suggest a possible role of H_2_O_2_ in TCTP induction during flooding stress.

### Phytohormone and polyamines response to flooding stress

**Figure 6:**
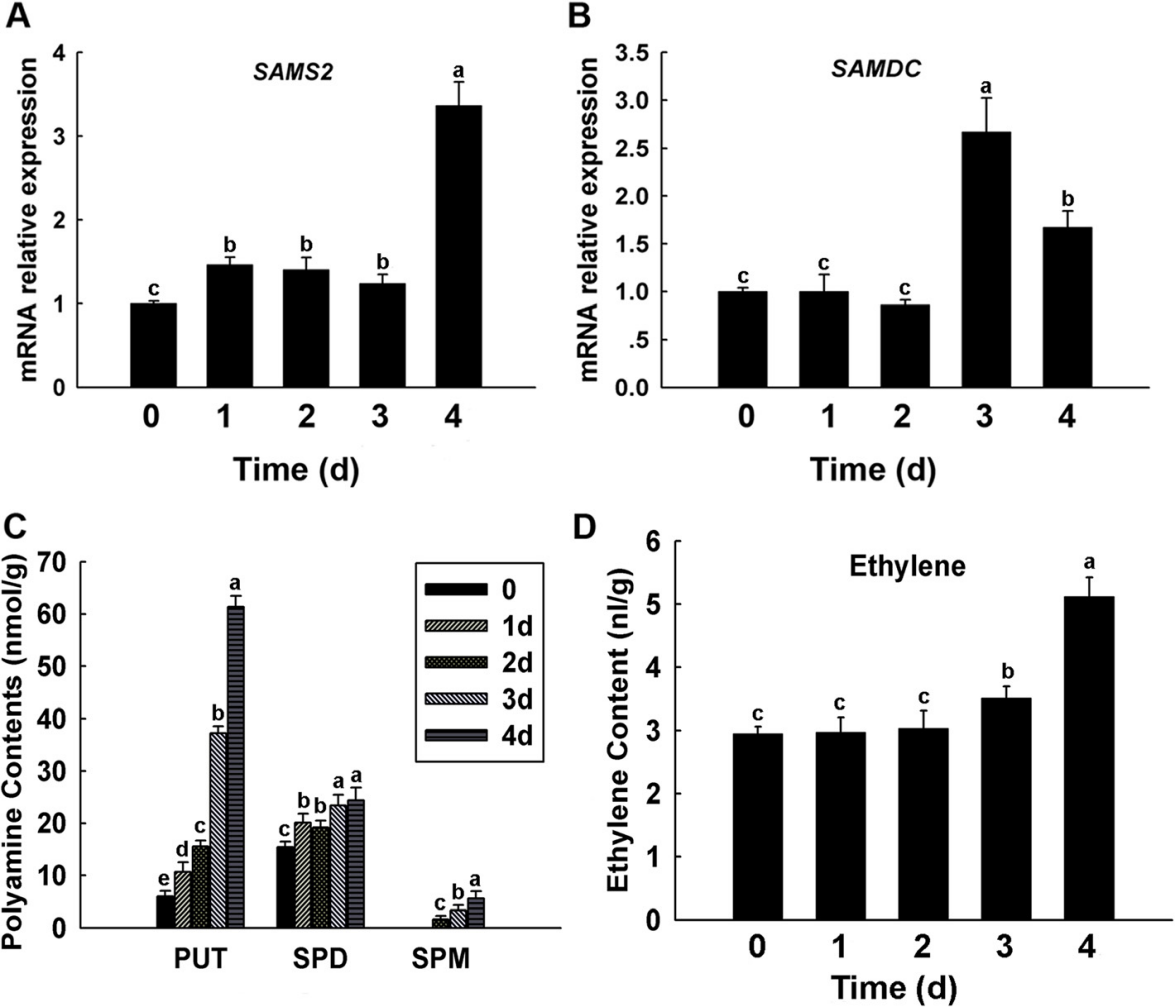
**Quantitative Real-time RT-PCR analysis of the mRNA expression levels of SAMS2, DAMDC and polyamines, ethylene contents in maize leaves under flooding treatment for 0, 1, 2, 3, 4 d.** qRT-PCR assays for quantitative analysis of *SAMS2***(A)** and *SAMDC***(B)** mRNA expression levels under flooding treatment. HPLC assays of polyamine content in maize leaves under flooding treatment; putrescine (PUT), spermidine (SPD) and spermine (SPM) **(C)**. Gas chromatograph (GS) assays of ethylene content in maize leaves under flooding treatment; Ethylene contents **(D)**. Data are displayed as mean values ± SD from three independent experiments. The same letter indicated no significant difference, and different letters indicated significant differences, as determined by Fisher’s protected LSD test (p < 0.05).

**Figure 7:**
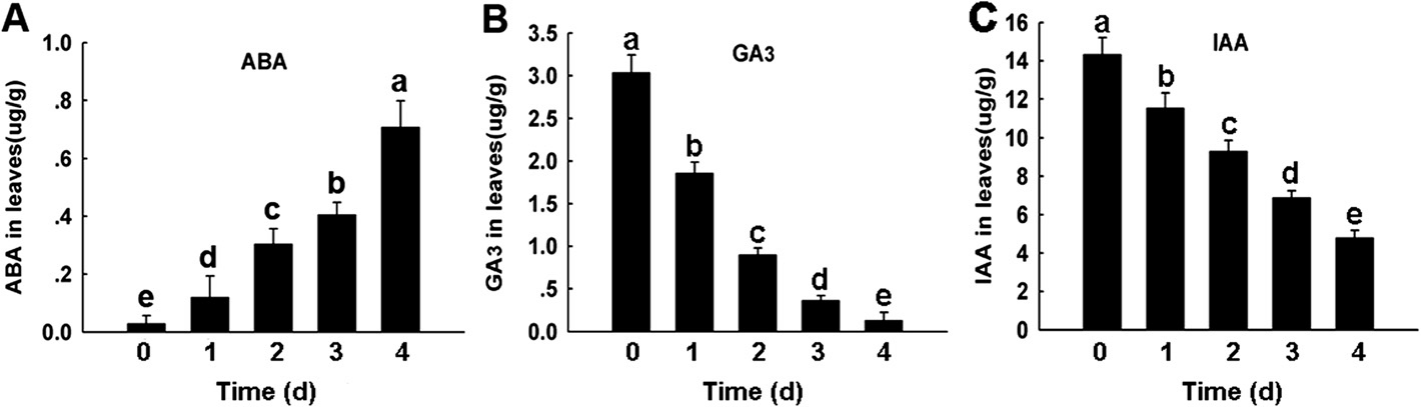
**Phytohormones contents in maize leaves under flooding treatment for 0, 1, 2, 3, 4 d.** UPLC assays of ABA, GA_3_ and IAA contents in maize leaves under flooding treatment. Data are displayed as mean values ± SD from three independent experiments. The same letter indicated no significant difference, and different letters indicated significant differences, as determined by Fisher’s protected LSD test (p < 0.05).

When under hypoxia tolerance, plant species elongate their petioles to reach the surface of the water and promote survival, with ethylene, auxin, abscisic acid, gibberellic acid and polyamines directing this signal transduction cascade [67,68]. Ethylene (ET) is a simple gaseous plant hormone involved in numerous biological process like leaf abscission, senescence, growth regulation, fruit ripening and many stress acclimations [69]. Polyamines are involved in a wide array of fundamental plant processes such as growth, development, senescence, membrane stabilization and adaptation to abiotic and biotic stresses [68]. In our study, we found the ethylene and polyamine levels to increased 4 days post-flooding treatment (Figure 6). S-adenosylmethionine synthase 2 (SAMS2, spot 952) is a key enzyme in the synthesis of S-adenosyl-L-methionine (SAM), a precursor for the biosynthesis of ethylene and polyamines [70], and was found to be up-regulated. SAM is converted into ethylene by ACC synthase (ACS) and ACC oxidase (ACO) [71]. Spermidine and spermine synthesis required decarboxylated S-adenosylmethionine (dcSAM), which is produced from SAM by the action of S-adenosylmethionine decarboxylase (SAMDC) [72]. However, in our study, the expression of SAMS2 and SAMDC increased after 4 days of treatment, with the expression of ACC synthase and ACC oxidase not established (Figure 6). Previous studies have found that under anaerobic conditions, ACC production is localized in the roots [73] and that ACO1 expression was lowest in the shoot [74]. Thus in the current study, SAMS2 was closely related to polyamines synthesis, while ethylene production was inhibited. Our study found the ethylene concentrations to increase, possibly due to ACC being transported from roots to leaves and converted into ethylene [73], while other papers have suggested that the xanthine oxidase-xanthine reaction reduces oxygen to both O_2_^-^ and H_2_O_2,_ which in the presence of transition metals generate hydroxyl radicals that act upon methional to release ethylene [75]. The leaf angle control protein was shown to be up-regulated, possibly because during flood condition plants angle the leaves upward to keep a portion of the leaves above water, with ethylene possibly promoting this response [76]. Additionally, ethylene regulates PISTILLATA-like MADS box protein expression, which is involved in ovary development and whose expression decreases following pollination [77]. In our study, ethylene expression increases in response to flooding stress and we hypothesis that ethylene play a role in inhibiting PISTILLATA-like MADS box protein expression during flooding. The gibberellin response element (GARE) of α-amylase promoters plays a central role in GA-regulated gene expression. HRT is a zinc-finger protein that binds the 21 nucleotide GARE and can repress GA-induced expression from the α*-Amy1* and α*-Amy2* promoters [26]. In our results, the expression of HRT was increased, while β-amylase and GA_3_ were decreased in the leaves during flooding treatment (Figure 7). Thus, flooding could inhibit GA synthesis in the roots, thus leading to a decrease in leaves, while the decrease in β-amylase expression may relate to feedback leading to accumulation of HRT. GA and ABA are antagonists and ABA could prevent the expression of amylase [78], with previous studies noting an increase in ABA expression during flooding conditions [79]. These findings were consistent with our results that noted an increase in ABA expression after 4 days of flooding treatment (Figure 7). Previous studies have found plants under flooding stress exhibit a decrease in GA expression and an increase in ABA expression, with reduced IAA concentrations noted in the leaves [80]. The present study, we also noted a decrease in IAA expression from the start of flooding (Figure 7). AUX/IAA protein is an active repressor, with its stability and activity modulated by auxin. As auxin concentrations eventually declined, AUX/IAA protein concentrations continue to climb until sufficient levels are reached to generate feedback [81]. However, in our study, the IAA concentration was lowest after 4 days of flooding treatment, with a decreased AUX/IAA protein expression also noted. It may be that as auxin concentrations eventually decline, that a delay occurs before AUX/IAA protein levels are seen to increase and then be impacted by feedback.

## Conclusion

In this study, we found PCD in the leaves of maize under flooding treatment and DIGE approaches to obtain a comprehensive proteomic description of flooding induced PCD was applied. A total of 32 differentially expressed spots were identified via 2D-DIGE, with 31 spots successfully identified by MALDI-TOF/TOF MS which represents 28 proteins. The identified proteins related to energy metabolism and photosynthesis, PCD, phytohormones and polyamines. All PCD related proteins exhibited an increased expression following flooding stress and TCTP was implicated as a potential PCD regulator in plants. Collectively, these findings shed light on flooding tolerance and show ability for Maize to restrict essential metabolites, thus reducing energy consumption, as a means to adapt to hypoxia. While the photosynthetic systems were damaged, the up-regulation of OEE1 appears to be able to maintain the PSII capacity during flooding stress. Furthermore, we observed an accumulation of H_2_O_2_ in leaves in response to flooding stress, thus suggesting a possible role of H_2_O_2_ in the induction of TCTP, whereas the photosynthetic decline may result in the ROS overproduction. Additionally, flooding induced a small amount of ethylene production, with a noted increase in SAMS2 expression relating to the accumulation of polyamines. This suggests that the accumulation of polyamines inhibited ethylene production to further delay senescence in maize leaves during flooding. Furthermore, the increased ethylene concentration may be due to ACC being transported from roots to leaves and converted into ethylene. In conclusion, this study lays the foundation for further investigations to enable the enhancement of flood tolerance in maize.

### Methods plant growth and flooding treatment

Maize seeds (*Zea mays* L.cv Nongda 108; from Nanjing Agricultural University, China) were sown on plastic plates and grown in a light chamber at 22°C to 28°C with a photosynthetic active radiation of 300 μmol m^-2^ s^-1^ and a photoperiod of 14/10 h (day/night) and watered daily. When the fourth leaves were fully expanded, they were subjected to flooding stress. To impose the treatment, seedling pots were in plastic containers (55 cm × 35 cm × 10 cm) and filled with water so that the water level was about 2 cm above the soil surface in the plant-containing pots [82], while control plants were appropriately watered during the experimental period. After 5 days of treatment, the 1^st^, 2^nd^, 3^rd^ and 4^th^ leaves from the bottom were sampled from both the treated and control plants.

### Physiological measurements

Each experimental treatment was performed in quadruplicate. Chlorophyll fluorescence parameters were initially taken on dark-adapted leaves for 30 min, using a chlorophyll fluorometer (FMS 1, Hansatech, Norfolk, UK) with an excitation soure intensity of 600 μmol m^-2^ s^-1^. The maximum quantum yield of PSII (Fv/Fm), the photochemical quenching coefficient (qP) and the non- photochemical quenching coefficient (qN) were calculated as described previously [83]. Individual leaves were removed from the steam and immediately weighed to determine the fresh mass (FM). To obtain the turgid mass (TM), leaves were floated in distilled water inside a closed petri dish. During the imbibitions period, after gently wiping the water from the leaf surface, leaf samples were weighed periodically, till constant. At the end of the imbibition period, leaf samples were placed at 80°C for 48 h to obtain the dry mass (DM). All mass measurements were made using an analytical scale (precision of 0.0001 g) and relative water content calculated by the equation: RWC (%) = [(FM ‒ DM)/(TM ‒ DM)] [84].

### Histochemical detection of H_2_O_2_

H_2_O_2_ was detected in the leaves of plants by using DAB as a substrate [85]. Plants were subjected flooding 4d, with the base of stem excised and a 1 mg mL^-1^ solution of DAB (pH 3.8) supplied for 8 h under light at 25°C. Following treatment, the leaves were decolorized by immersion in boiling ethanol (96%) for 10 min. This treatment decolorized the leaves while leaving the deep brown polymerization product produced by the reaction of DAB with H_2_O_2_. After cooling, the leaves were photographed.

### DNA laddering analysis

Half gram of flood treated or control leaves were ground into powder with liquid nitrogen, transferred into extraction buffer (100 mM Tris–HCl, pH 8.0, 20 mM EDTA, 1.4 M NaCl, 2% CTAB and 0.2% β-mercaptoethanol) and incubated at 65°C for 1 h. Then DNA was extracted with phenol/chloroform/isopropanol (25:24:1 by volume) and precipitated with isopropanol for 30 min. Pellets were washed with 70% ethanol, dissolved in TE buffer (10 mM Tris–HCl, 1 mM EDTA at pH 7.4) and treated with DNase-free RNase A to digest any remaining RNA. Finally, 10 μg of DNA from each sample was separated by electrophoresis on a 2% agarose gel [86].

### Preparation of total protein extract

Protein was extracted using Mg/NP-40 extraction buffer [87]. The leaves were placed in liquid nitrogen, transferred to a pre-chilled mortar and ground with a pestle in liquid nitrogen to a fine powder. The powder was homogenized in 10 ml of ice-cold Mg/NP-40 extraction buffer containing 0.5 M Tris–HCl, 2% v/v NP-40, 20 mM MgCl_2_, 2% v/v β-mercaptoethanol, 1 mM phenylmethylsulfonyl fluoride (PMSF) and 1% w/v polyvinylpolypyrrolidone (PVPP) at pH 8.3. After centrifugation at 13000 × g for 20 min at 4°C, proteins were precipitated from the supernatant by adding six volumes of cold acetone at -20°C overnight. After centrifugation at 13000 × g for 20 min at 4°C, pellets were washed with ice-cold acetone containing 0.1% w/v DTT and centrifuged again, with the washing procedure repeated three times. Pellets were finally freeze-dried re-suspended in labeling buffer (30 mM Tris, 7 M urea, 2 M thiourea and 4% w/v CHAPS at pH 8.5). Protein content was determined via Bradford method with a bovine serum albumin standard to ensure a protein concentration between 5–10 mg/ml.

### CyDyes fluorescence protein labeling and 2D-DIGE

Leaf extracts were prepared and resolved by 2D-DIGE. Four gel analyses were performed according to the principles of experimental design in DIGE [88]. Each gel contained one control sample labeled with Cy3 fluorescent dye, one treated sample labeled with Cy5 fluorescent dyes and an internal standard containing equal amounts of all samples and Cy2 labeled. The CyDye DIGE Fluor minimal dye of DMF was reconstituted to make a stock of 1 nmol/μl and a subsequent working solution of 200 nmol/μl. Mixed protein samples and fluorescent dyes (50 μg: 400 pmol) were incubated for 30 min on ice in the dark. The labeling reaction was ended by the addition of 10 mM lysine for 10 min. The three labeled samples were mixed to contain 50 μg of Cy3/Cy5-labeled samples and 50 μg of Cy2-labeled internal standard and adjusted with rehydration buffer (8 M urea, 4% w/v CHAPS, 2% DTT and 2% v/v pH 3–10 pharmalyte) to a final volume of 250 μl. The samples were separated in the first dimension by isoelectric focusing (IEF) at 20°C using an IPGphor3 isoelectric focusing system (GE Healthcare). The 13 cm 4–7 IPG strips (GE Healthcare) were incubated overnight with protein samples in rehydration buffer at room temperature. Isoelectric focusing was performed for 6 h reaching a total of 20 kVh. After IEF, the strips were transferred to equilibration buffer (6 M urea, 50 mM Tris–HCl (pH 8.8), 30% glycerol and 4% SDS) supplemented with either 1% (w/v) DTT or 2.5% (w/v) iodoacetamide for 15 min at room temperature. The IPG strips were then placed onto 12% polyacrylamide gels and overlaid with 0.5% agarose in SDS-PAGE running buffer. Gel electrophoresis was carried out at 20°C in the Ettan Dalt six systems (GE Healthcare) at 30 mA per gel for 5 h and gels were imaged on a Typhoon 9400 Variable Mode Imager (GE Healthcare). Excitation/emission wavelengths for Cy2 (488/520), Cy3 (532/580) and Cy5 (633/670 nm) were analyzed with DeCyder 7.0 software (GE Healthcare). The fold change threshold was set at 2.5-fold to be considered significant. Moreover, spots with a p-value < 0.05 following a Student’s t-test comparing treated plants log standardized abundance values to control values were also considered significance.

### In-gel digestion and mass spectrometry

Protein spots were excised from preparative 2D-gels that had been stained with Coomassie brilliant blue. Spots were destained in 50 mM ammonium bicarbonate/50% methanol (v/v) in water, followed by 75% acetonitrile (v/v) for dehydration. Spots were then rehydrated with trypsin digestion solution overnight (20 h) at 37°C. Digested peptides were extracted using extraction buffer (50% Acetonitrile, 0.5% Trifluoroacetic Acid (TFA)) and dried by vacuum centrifugation. Peptides were dissolved in 0.1% TFA, desalted with a C18 ZipTip (Millipore, Bedford, MA), mixed with 6 mg/ml a-cyano-4-hydroxy-cinnamic acid in 50% acetonitrile and 0.1% TFA and spotted onto MALDI target plates. Mass spectrometry was performed on a Bruker-Daltonics AutoFlex TOF/TOF LIFT Mass Spectrometer (Bruker Daltonics, Bremen, Germany) operated in reflectron mode. Database searches with both peptide mass fingerprinting (PMF) and MS/MS were performed using the MASCOT program. The database was set to the National Center for Biotechnology nonredundant (NCBInr) (updated on September 4, 2012), which contained 19,737,474 sequences. The other parameters for searching were enzyme of trypsin, one missed cleavage, fixed modifications of carbamidomethyl (Cys), peptide mass tolerance of 100 ppm, fragment mass tolerance of ± 0.5 Da, peptide charge state of 1+ and monoisotopic. Only significant hits, as defined by the MASCOT probability analysis (p < 0.05) were accepted.

### Dark treatment of leaves in *Arabidopsis thaliana* and rice and heat shock treatment of rice protoplasts and TMV infected tobacco leaves

Green leaves from *Arabidopsis thaliana* and rice were placed onto dishes containing distilled water under continuous dark at 23°C [89]. Heat shock was induced in rice protoplasts using a water bath at 48°C for 15 min, with samples returned to 28°C for recovery [62]. TMV infected tobacco leaves were rubbed with viral suspensions (1 μg/ml) mixed with 20 mg/ml carborundum carmine in water on to the leaf lamina. After abrasion, carborundum carmine was washed by spraying with water and the tobacco was allowed to grow in greenhouse [90].

### Polyamines concentration determination

Leaf samples (1 g) were fully ground in liquid nitrogen, soaked in 3 ml of 0.6 M HClO_4_ for 1 h on ice, centrifuged for 20 min (17,000× g, 4°C) and the 1 ml supernatant mixed with 14 μl of benzoyl chloride. 2 ml of NaOH (2 M) was added to each samples followed by vortexing for 20 s, a 20 min incubation at 37°C and the addition of 2 ml of saturated NaCl. Benzoyl-polyamines were extracted in 2 ml of diethyl ether. After centrifugation at 3000 × g for 15 min, 1 ml of the ether phase was collected, evaporated to dryness in a vacuum concentrator and redissolved in 100 μl of methanol. Polyamine standards were processed in a similar way to benzoylate, with 10 μl aliquots of each redissolved sample were injected into a Liquid Chromatograph. The samples were eluted through a 150 × 3.9 mm, 4 μm particle size C18 reverse-phase column at a flow rate of 0.7 ml/min. The detection wavelength was 230 nm and the column was held at 30°C [91].

### Ethylene concentration determination

The third leaves of the control and flooding treatments of maize were confined in 25 ml sealed glass flasks without agitation. In order to prevent any stress, incubation conditions were the same as culture conditions. After a 24 h dark incubation, at 28°C, 1 ml of gas was taken from the flask with a gas-tight syringe and analyzed using gas chromatograph (GC). An Al_2_O_3_ (30 m × 0.53 mm × 1.5 μm) capillary column was used with a carrier gas (N_2_) and flow rate of 48 ml/min. The spray, column and detector temperatures were maintained at 130°C, 40°C and 220°C respectively [92].

### GA, IAA, ABA concentration determination

Tissue samples (0.5 g fresh weight) were ground to a powder under liquid nitrogen and soaked in 5 ml of 80% methanol at 4°C for 12 h, centrifuged for 15 min (14000 rpm) and the supernatant collected, with the centrifugation and supernatant collection repeat twice. The samples were concentrated to 5 ml under nitrogen gas, passed through Sep Pak C^18^-cartridges and the hormones eluted with 80% methanol. The eluates were then dried under nitrogen gas, extracted in 1 ml of 60% methanol in 1% acetic acid in distilled water and passed through a 0.45 μm filter prior to loading. The hormones were then analyzed by ultra-performance liquid chromatography (UPLC) using mobile phase A (1% acetic acid in distilled water) and B (methanol) to give A: B = 6: 4. The column was held at 35°C with a flow rate of 1.0 ml/min and a detection wavelength of 260 nm [93].

### Quantitative real-time RT-PCR (qRT-PCR) analysis

Total RNA was prepared from various samples with TRIZOL reagent (Invitrogen) according to the manufacturer’s instructions. Cyclophilin, 18S rRNA, actin and eEF1α were used as internal controls for the normalization of maize, rice, *Arabidopsis thaliana* and tobacco respectively [94-97]. Primers were designed for qRT-PCR analysis (Table 2) and all of the RNA samples were diluted to 200 ng/μl. Expression levels were evaluated using a two-step qRT-PCR kit with SYBR®Green (Takara) with a final volume of 20 μl (10 μl SYBR®Green qPCR Mixture, 10 μM forward and reverse primers) in a 7500 Real time PCR System (ABI). All reactions were performed in three biological replicates. The threshold cycle values (Ct value) of the genes and internal control genes for the different samples were calculated by the 2^-ΔΔCT^ method, and the mean ± SD should always be calculated after the 2^-ΔΔCT^ transformation in order to perform statistical analysis [98].

**Table 2:** Specific primers used for RT-PCR analysis

**Gene**	**Forward primer**	**Reverse primer**
ZmCyclophilin (*Zea may*)	5’-atcgtgatggagctgtacgccaa-3’	5’-tggcacatgaacacggggat-3’
ZmTCTP (*Zea may*)	5’-cactaatccgacattcctcta-3’	5’-aacaagcaatccaatcttgg-3’
zm50s RPL (*Zea may*)	5’-ggttgtgagatgttgtat-3’	5’-aagaaggatgtatatgttgt-3’
ZmPH (*Zea may*)	5’-gctacttacgcagagaat-3’	5’-caccagagttccacataa-3’
zmDREPP4 (*Zea may*)	5’-gaaggaagaggaggataagc-3’	5’-ttaccagcagcaagcaag-3’
zmFpPKIWI502 (*Zea may*)	5’-actactgaacattacatc-3	5’- taatcatctcatctcctt-3
zmHSP70 (*Zea may*)	5’-aggaggtggactaagcggat-3	5’-cttaaaacgcgtgccacgat-3
ZmADK (*Zea may*)	5’-agaaggtcctcccgtatgct-3	5’-tgcctgaagccaaaggaagt-3
ZmMDH (*Zea may*)	5’-cagcggaatgcatttgcca-3	5’-tgcatcatagtcaaattcgtgtgg-3
zmOEE1 (*Zea may*)	5’-gaccgccgtcatggatctt-3	5’-aaaagcgacagcccgaat-3
ZmPGK (*Zea may*)	5’-gcgctagcctggaattgttg-3	5’-tcggtagcagacctccgtaa-3
ZmPEPCK (*Zea may*)	5’-aaacgatggtgtgtgtgcgt-3	5’-acaagaccagagaccagacg-3
ZmFbp (*Zea may*)	5’-ctccaacgaggtgttctcca-3	5’-gaacacgacgatgtagttgcc-3
zm3-Bhsd (*Zea may*)	5’-gacgctgtcggagaaccatt-3	5’-cagggggtgcaaggattagg-3
zmHrBP1 (*Zea may*)	5’-gctcagggtgtttgtcat-3	5’-aggaagaaggatggcaatc-3
zmChitinase (*Zea may*)	5’-gtcaccaacatcatcaac-3	5’-caagcaagtcacagtatc-3
18S rRNA (*Oryza*)	5’- cctatcaactttcgatggtaggata-3’	5’-cgttaagggatttagattgtactcatt-3’
TCTP (*Oryza*)	5’-ttcctttacttctctcat-3’	5’-tcatctcaacatccataa-3’
eEF1α (*Nicotiana*)	5’-taatgtggttcttagttc-3’	5’-agttccgaattaagtatc-3’
*hsr203j* (*Nicotiana*)	5’-cagagttcatcaacaagcatta -3’	5’-acaatcaagacggtacatca-3’
TCTP (*Nicotiana*)	5’-tcaaggagcaaccaagta-3’	5’-caccattccagtatcatcag-3’
Actin (*Arabidopsis thaliana*)	5’-gccatccaagctgttctctc-3’	5’-gctcgtagtcaacagcaacaa-3’
TCTP (*Arabidopsis thaliana*)	5’-ctgtgttggaagattctca -3’	5’-agattcgggagtttaatttaga -3’
HRT (*Zea may*)	5’-aagaacctcagaggcaaagc-3’	5’-acattctcccagaaggctgc-3’
CLA (*Zea may*)	5’-gctgactccgactttgacga-3’	5’-ctgctttcctgtcgtccctt-3’
SAMS2 (*Zea may*)	5’-ccctttcggtgttcgtgga-3’	5’-acagcacagcactgcaacat-3’
zm-Plmbp (*Zea may*)	5’-ctttgctgggctgatttccg-3’	5’-tcttgccttcgtctcgcaat-3’
IAA/AUIX (*Zea may*)	5’-ttacagtccagagcaaga-3’	5’-tacagaccataaggcagaa-3’
ACS6 (*Zea may*)	5’-agctgtggaagaaggtggtcttcgaggt-3’	5’-agtacgtgaccgtggtttctatga-3’
ACO15 (*Zea may*)	5’-ctcgtcttcgatcaattcccaagt-3’	5’-tacattatcattatttctccggctgt-3’
SAMDC2 (*Zea may*)	5’-gaaaggcacttggtgcagag-3’	5’-ccgtcaaagcagtggaaaaca-3’

### Statistical analysis

Statistical procedures were carried out with the software package SPSS 10.0 for Windows. The means were considered to be significantly different by Fisher’s protected LSD test at p < 0.05.

## Abbreviations

2D-DIGE: Two-dimensional fluorescence difference gel electrophoresis; ABA: Abscisic acid; ACS: 1-aminocyclopropane-1-carboxylate synthase; ACO: 1-aminocyclopropane-1-carboxylate oxidase; AMP: Adenosine monophosphates; CHAPS: 3-[(3-cholamidopropyl)dimethylammonio]-1-propanesulfonate; DAB: 3,3-diaminobenzidine; DMF: Dimethylformamide; DREPP 4: Developmentally regulated response 4; DTT: Dithiothreitol; ET: Ethylene; GA: Gibberellin acid; GARE: Gibberellin response element; GC: Gas chromatograph; HR: Hypersensitivity; HrBP1: Harpin binding protein 1; IAA: Indole acetic acid; LSD: Least significant difference; MALDI: Matrix-asscisted laser desorption/ionization; OEE1: Oxygen-evolving enhancer protein 1; PCD: Programmed cell death; PMF: Fingerprinting; PMSF: Phenylmethylsulfonyl fluoride; PPV: Plum pox virus; PSII: Photosystem II; PVPP: Polyvinylpolypyrrolidone; PUT: Putrescine; qRT-PCR: Quantitive real-time PCR; ROS: Reactive oxygen species; RWC: Relative water content; SAM: S-adenosyl-L-methionine; SAMDC: S-adenosylmethionine decarboxylase; SAMS2: S-adenosylmethionine synthase 2; SPD: Spermidine; SPM: Spermine; TCA: Trichloroacetic acid; TCTP: Translationally-controlled tumor protein; TFA: Trifluoroacetic acid; TOF: Time-of-flight; UPLC: Ultra-performance liquid chromatography.

## Competing interests

The authors declare that they have no competing interests.

## Authors’ contributions

CY carried out all experiments in addition to the detection of DNA ladder. WHJ carried out the detection of DNA ladder. CX, BYQ, ZW conceived designed and coordinated this study. All authors read and approved the final manuscript.
